# Monitoring of Torque Induced Strain in Composite Shafts with Embedded and Surface-Mounted Optical Fiber Bragg Gratings

**DOI:** 10.3390/s21072403

**Published:** 2021-03-31

**Authors:** Maria Konstantaki, Georgios Violakis, Georgios A. Pappas, Thomas Geernaert, Nikos Korakas, Nikos Tiriakidis, Thomai Tiriakidi, Kosmas Tiriakidis, Hugo Thienpont, Francis Berghmans, John Botsis, Stavros Pissadakis

**Affiliations:** 1 Foundation for Research and Technology-Hellas (FORTH), Institute of Electronic Structure and Laser (IESL), Heraklion, 70013 Crete, Greece; vgeo@iesl.forth.gr (G.V.); nkorakas@iesl.forth.gr (N.K.); pissas@iesl.forth.gr (S.P.); 2 Ecole Polytechnique Fédérale de Lausanne (EPFL), LMAF, STI, CH-1015 Lausanne, Switzerland; gpappas@ethz.ch (G.A.P.); john.botsis@epfl.ch (J.B.); 3 Brussels Photonics (B-PHOT), Department of Applied Physics and Photonics, Vrije Universiteit Brussel and Flanders Make, Pleinlaan 2, 1050 Brussels, Belgium; thomas.geernaert@vub.be (T.G.); hugo.thienpont@vub.be (H.T.); Francis.Berghmans@vub.be (F.B.); 4 BT Composites S. A., 53100 Florina, Greece; nikos@btcomposites.gr (N.T.); thomai@btcomposites.gr (T.T.); kosmas@btcomposites.gr (K.T.)

**Keywords:** carbon fiber reinforced polymer, optical fiber Bragg gratings, strain monitoring, torsion, structural health monitoring, optical fiber sensors

## Abstract

In this study, silica glass, optical fiber Bragg gratings (FBGs) are used for torque-induced strain monitoring in carbon fiber reinforced polymer (CFRP) hollow shafts toward the development of a methodology for structural load monitoring. Optical fibers with gratings are embedded during shaft manufacturing, by an industrial filament winding process, along different orientations with respect to its central axis and surface mounted after production. Experimental results are supported by numerical modeling of the shaft with appropriate boundary conditions and homogenized material properties. For an applied torque up to 800 Nm, the strain sensitivity of an embedded grating positioned along the reinforcing fibers’ direction winded under 55° is in the order of 3.6 pm/Nm, while this value is more than 4× times higher than the other examined orientations. The study also shows that surface-mounted optical fiber Bragg gratings along the reinforcing carbon fibers’ direction perform equally well in monitoring strains in composite shafts under torque.

## 1. Introduction

A recurrent objective in many application fields, including aerospace, automotive, and marine, is the use of structures that are lightweight and easy to fabricate while exhibiting enhanced performance in terms of durability, fatigue resistance, and consistent operation. Carbon fiber reinforced polymer (CFRP) composite materials constitute a favorable solution due to their superior strength-to-weight ratio, ease of fabrication of complex shapes, and tailored properties [1]. Polymer matrix composites can be fabricated by different techniques such as autoclave, filament winding, and resin transfer molding. The choice of process is governed by criteria such as product shape and size, characteristics of reinforcements, and end-use specifics. In particular, filament winding [2] is an extensively used technique that allows the production of hollow structures of cylindrical geometry that find application as torque transmission shafts, process pipes, and pressure vessels. 

A shortcoming of composites is that due to their complex structure, even small manufacturing flaws can lead to damage and abrupt failure once exposed to their typical harsh operation conditions. Structural health monitoring (SHM) is of paramount importance in composite structures for flaw detection and determination of the remaining strength and lifetime of the structure [3]. Deformation monitoring, an essential part of SHM that provides spatially resolved information about loads applied to the structure, can be carried out employing a network of sensors integrated within, or onto, the structure. Optical fiber sensors (OFS) constitute a favorable type of sensor, ideally suited for the task since they can be embedded in the composite with minimal intrusion owing to their small size and weight [4]. In a different approach, OFS can be surface-mounted on the composite to minimize concerns related to modifications of the production process, the fragility of the fiber at the composite egress point, and sensor replacement. 

In recent years, optical fiber Bragg gratings (FBGs) have been proposed for real-time assessment and control of the manufacturing process of composite structures [5,6,7,8,9] and structural health monitoring during subsequent lifetime operation in various applications [10,11,12]. Specifically, for filament wound structures, researchers have embedded FBGs to monitor residual strains during the curing process [13] and have tested the response of the sensors under hydrostatic pressure loading in view of underwater applications [14]. Reports also exist on the use of FBGs externally attached on the domes and cylinder of a filament wound composite pressure vessel, to monitor pressure-induced strain [15], while in a different study, impact damage detection was achieved using embedded FBG sensor arrays [16].

In this study, we report on the use of optical fiber FBGs in filament wound composite shafts for monitoring torque-induced strain. Rotating machinery constitutes a critical component in numerous applications as parts of systems such as transportation vehicles, generators, and gearing equipment and plays an important role in modern industrial development. Torque load creates a shear strain distribution over the cross section of a structure, and for a safe operation, its maximum allowable torsional strain should not be exceeded. Operation of shafts under extreme conditions, such as abrupt acceleration/deceleration, continuous operation under high torque loads, etc., can induce failure due to buckling [17]. The use of the proposed strain monitoring system could serve the quality assurance process after production by quantifying the strain at particular torque levels.

Conventionally, torque in a rotating shaft can be measured directly by in-line torque transducers [18], which, however, suffer from high cost, may induce unwanted vibration to the system, and usually can accommodate only limited torque ranges. Indirect torque measuring approaches rely, mainly on the measurement of surface strain and subsequent torque calculation [19]. These systems offer the advantage of direct mounting of the sensors (usually strain gauges) on the shaft without the need for shaft modification or disassembly while exhibiting good accuracy at a relatively low cost. FBGs perform very well as strain sensors, and their use for torque measurements in aluminum tubes has been previously demonstrated by Gao et al. [20], with externally attached FBGs, in which the effect of sensor orientation was also discussed. Furthermore, Leal-Junior et al. [21] have incorporated polymer FBG arrays [22,23,24] in a series elastic actuator’s spring for angle, quasi-distributed displacement, and torque sensing.

Herein, we report, for the first time to the best of our knowledge, the use of embedded and surface-mounted FBG sensors to measure the torque-induced strain in a composite shaft that, compared with conventional metal shafts, constitutes a complex, non-uniform, layered structure. Toward this goal, we fabricated hollow CFRP shafts, equipped with embedded and surface-mounted FBG sensors, and measured their strain response when the composite was subjected to increasing levels of static torque. FBGs were embedded during filament winding of the structure, employing different sensor orientations with respect to the shaft axis (longitudinal, parallel to the reinforcing fibers, and circumferential) to examine the effect of sensors’ orientation on their performance and applicability of FBG installation during a standard industrial winding process. Furthermore, we surface-mounted FBGs onto the composite, after production, to compare the response of embedded and external FBG sensors in the monitoring of torque-induced strain. Numerical studies were also carried out, assuming a homogenized material model, to predict the mechanical behavior of the composite shafts under torsion. The experimental data and pertinent numerical simulations are analyzed and discussed with respect to sensor orientation and incorporation method, i.e., embedment or surface mounted. The results, provide useful information on the use of FBGs toward an efficient SHM technique of the aforementioned structure group, such as axles and shafts, subjected to torsion.

## 2. Materials and Methods

The FBGs used in this study were inscribed employing a standard phase mask setup with a 193 nm high spatial coherence and 10 ns pulse duration excimer laser (Braggstar, TUI laser) [25]. Gratings of 5 mm length and transmission strength of ~10 dB were inscribed in Nufern GF1B photosensitive single-mode low-NA fiber with a laser pulse energy density of 160 mJ/cm^2^. Phase masks with different pitches were used, resulting in FBGs with reflection signal at distinctive Bragg wavelengths, *λ*_B_, in the 1535–1555 nm range, to facilitate multiplexed interrogation of sensors along a shaft. The FBGs were annealed post-inscription at a temperature of 250 °C for conditioning prior to the thermal curing process.

**Figure 1:**
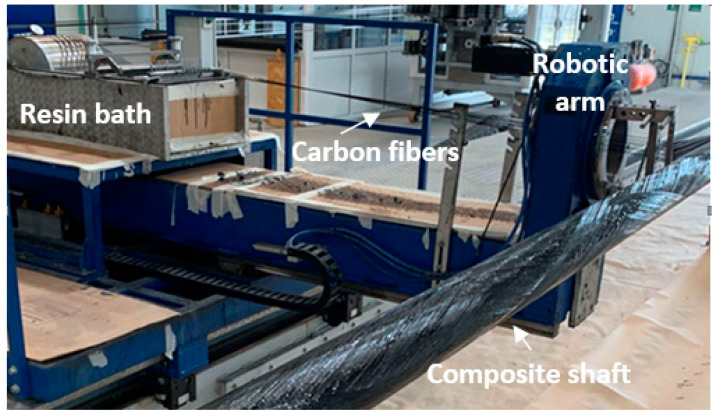
Filament winding system for fabrication of carbon fiber-reinforced polymer (CFRP) shafts.

CFRP cylindrical shafts were fabricated at the premises of B&T Composites S.A., Florina, Greece, using a filament winding system (see Figure 1). Under the filament winding technique, a robotic arm applied resin-impregnated carbon fibers onto a spinning metal mandrel at a specific inclination angle for each layer following a predefined layup. The resin was then cured at a maximum temperature of 150 °C, while the structure was continuously rotated along its longitudinal axis to ensure even resin content. Toray T700S 150gsm carbon fiber unidirectional (UD) filaments were used, impregnated with the VORAFORCE™ filament winding System (TW 100-Epoxy/TW 105-Hardener/TC 3000-Catalyst) from Dow Chemical Company (Midland, Michigan, United States). A 2.2 m long shaft was fabricated with 6 cross-ply carbon fiber layers at ±55° (one filament wound layer is composed of two UD layers) and 2 reinforcing hoop layers at 86° with the following sequence: ±55°, ±55°, +86°, ±55°, ±55°, +86°, ±55°, ±55°. The applied 67% fiber mass content provides a nominal cured ply thickness of ~145 µm with a nominal fiber volume fraction, *V_f_*~57.5%. Based on the layup design, the external diameter of the shaft was 49.5 mm, and the nominal wall thickness 2.0 ± 0.2 mm. These values were verified after testing by local thickness measurements on sectioned samples described in Section 4.1.

Optical fibers were embedded manually by briefly pausing the winding process and rotation of the shaft. The sensors were positioned at the desired location/orientation and were manually held in place momentarily, until the next layer of carbon fibers was applied by the machine, to secure the embedment of the sensors. For an optimal strain transfer between the fiber and structure, the standard acrylate fiber coating was stripped at a length of about 50 mm around the inscribed FBGs. The egress of the fiber was through the outer surface of the composite structure, and due to being the most vulnerable point of the sensor, it was reinforced using Polytetrafluoroethylene (PTFE) tubing (Outside Diameter-OD 0.6 mm). Following the embedment of all different sensors, a number of lead-out fibers required careful intervention from the system operator to ensure proper egress from the composite. Under a more automated, future fabrication approach the optical fibers could be mechanically applied by the robotic arm along with the carbon fibers.

FBGs were embedded at three different orientations—parallel to the shaft axis (0° angle with respect to the shaft axis, longitudinal orientation); parallel to the direction of the reinforcing fibers (55° angle with respect to the shaft axis, helical orientation); and circled around the shaft (90° angle with respect to the shaft axis, circumferential orientation). Additional FBGs were embedded at each orientation to increase survival potential; however, only one FBG per orientation was considered in the subsequent torque monitoring study due to spectral span limitations of the interrogation system. Following curing of the composite shaft, the metal mandrel was extracted and additional FBGs were surface-mounted, using a two-component adhesive (X120, HBM) optimized for optical fiber installation applications [11].

**Figure 2:**
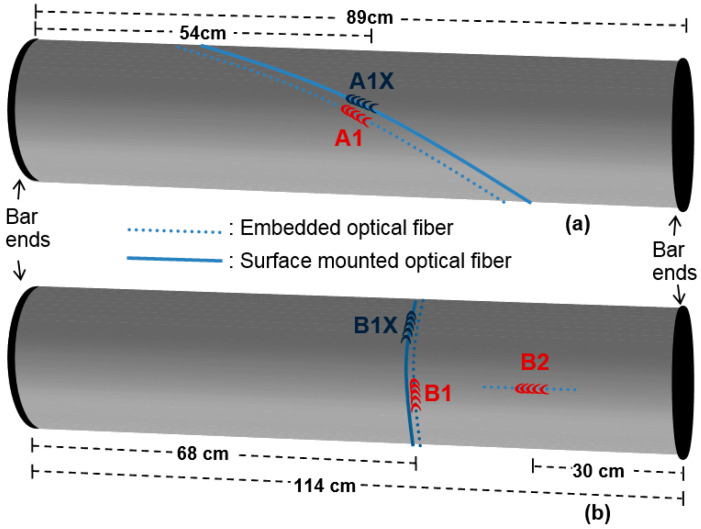
Location and orientation of fiber Bragg grating (FBG) sensors along (**a**) shaft A, (**b**) shaft B. Surface-mounted gratings (A1X, B1X) are illustrated in blue, while embedded gratings (A1, B1, B2) are in red. The shaft’s nominal diameter is 49.5 mm; the schematic is not to scale.

For the purpose of the experimental study related to the monitoring of torque induced strain, the composite shaft described above was cut with a diamond-coated disk to produce two samples identical in terms of diameter and carbon fiber layup and with comparable length (shaft A: 89 cm and shaft B: 114 cm, respectively). The orientation of the embedded and surface-mounted FBGs of the two shafts that were monitored under torsion is illustrated in Figure 2. Shaft A was equipped with an embedded (A1) and a collocated surface-mounted FBG (A1X), while both FBGs were positioned along the reinforcing fibers’ direction (55°). Shaft B had an embedded grating (B1) placed circumferentially (90°) and a corresponding externally mounted FBG (B1X). Grating B1X was at the same axial location as grating B1; however, since it was identified post-fabrication, the two gratings were placed 2 cm apart on the perimeter of the shaft, which corresponds to a rotation angle of 45°. Shaft B was also equipped with an FBG embedded longitudinally (B2).

**Figure 3:**
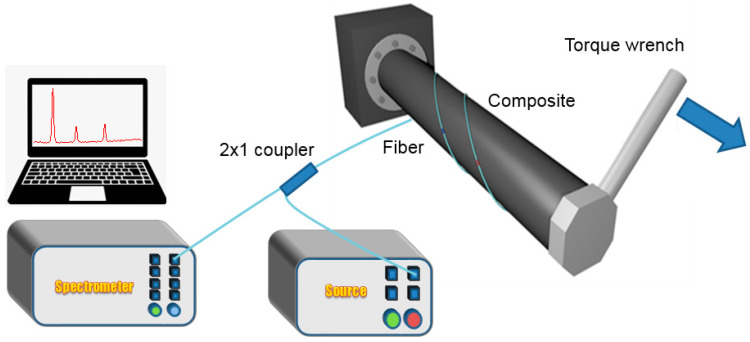
Illustration of torque-induced strain monitoring experimental setup with a torque wrench.

To facilitate testing of the shafts during the experimental investigation, chromium–vanadium steel bar ends were attached at the shaft edges using heat-curable resins. Through these metallic fittings, one end of the shaft was securely clamped, while torsion was applied to the other one using a torque wrench (Utensilgeat 1100). The response of the sensors in monitoring torque-induced strain was studied by applying a controlled static torsion to the shafts and recording the reflected spectral signal evolution of the gratings (see Figure 3). Further validation of the reported technology in rotating shafts would be possible using commercially available fiber optic rotary joints. The torque wrench was set to pre-defined torque values, and loading was then manually applied to the shaft with increasing steps of 50 or 100 Nm, up to a maximum value of 800 Nm with a waiting time of a few minutes between each loading. As mentioned already, the FBGs of each shaft were wavelength-multiplexed to allow simultaneous interrogation. This load evaluation system is not suitable for loading-unloading cycles, therefore hysteresis is not investigated.

The FBG reflection spectra were acquired in real time at a 10 Hz acquisition rate using an Ibsen IMON 256USB FBG spectrometer and a 2 × 1 coupler. Since the reflected intensity spectra showed no birefringence of the Bragg peaks, maximum Bragg peak wavelengths were identified with a peak fitting algorithm, in order to determine the wavelength shifts, Δ*λ_B,i_* per ith FBG. The use of the peak fitting algorithm was employed in order to enhance the effective resolution of the FBG interrogator, which has a hardware resolution of 0.2 nm. The FBGs fabricated for these measurements had on average a 1/e^2^ width of 0.6–0.8 nm, resulting in spectra consisting of 4–7 measurement points, including the points outside the 1/e^2^ width. The produced FBGs had a slightly apodized top-hat profile, and their spectral shape could be accurately approximated using a Gaussian function of the form: $\;f\left(x\right)=Aexp\left[-{\left(x-x_{c}\;\right)}^{2}/2\sigma^{2}\;\right]$, where A and *σ* are constants that depend on grating amplitude and width and x_c_ corresponds to the Bragg wavelength peak. The measured FBGs were fitted to the Gaussian function, and the Bragg wavelength was determined by the *x_c_* value output of the fitting process. This method benefits by using all the experimentally measured points to fit the Gaussian function and therefore provides higher accuracy in determining the Bragg peak, compared to single-point measurement. Based on the measured wavelength shifts, Δ*λ_B,i_* per ith FBG, corresponding normal axial strains were calculated assuming $\varepsilon_{z,i}=\left(\Delta \lambda_{Bi}/\lambda_{B,i}\right)\cdot \left(1/k\right)$, (*z*: optical fiber axis’ direction) with the gauge factor *k* = 0.789 [26]. During all measurements, the environmental temperature was recorded to ensure that the FBG sensor wavelength shift due to temperature change was negligible. Considering a maximum variation in room temperature of the order of 1 °C, applied during the torque study cycle, the corresponding wavelength shift of the FBGs is approximated at 10 pm [27], thus resting well within the experimental error acknowledged in the measurements. For future applications, appropriate FBG temperature compensating techniques [28] could be employed to eliminate temperature crosstalk.

## 3. Mechanical Analysis

### 3.1. Material Homogenization

In order to obtain the homogenized cured ply properties, the conventional rule of the mixture was considered [29], using the nominal $V_{f}$. The Young’s modulus of the epoxy-based on the manufacturer is $E_{m}=$ 3.0 GPa [30], while a typical Poisson’s ratio $\nu_{m}=\;$0.35 was considered, providing a shear modulus $G_{m}≅$ 1.1 GPa for the elastic isotropic case. The nominal tensile fiber modulus provided by the manufacturer is $E_{11,t}=$ 230.0 GPa [31]. The longitudinal UD lamina modulus, $E_{11}$, was evaluated using the rule of mixtures as $\;E_{11}=V_{f}\cdot E_{11,t}+\left(1-V_{f}\right)\cdot E_{m}$, using the nominal *V_f_* = 57.5% (see also Section 2). The remaining engineering constants of the lamina were approximated with Halpin–Tsai micromechanical semi-empirical corrections [29] ($\xi_{1}=2\;$for $E_{22}$, $\xi_{2}=1$ for $G_{12}$), with the remaining fiber properties obtained by [32]. The corresponding calculated constants are listed in Table 1.

Based on microscopy examination, which will be presented in Section 4.1, slight realignment of the reinforcing fibers, attributed to the compaction during the curing process, prevents a distinct consideration of each ply as an oriented lamina at the numerical modeling scheme. Instead, material homogenization, based on UD lamina constants was adopted, using the classical laminate theory (CLT) [30] for the in-plane properties (1′–2′ plane). The remaining through-thickness properties (1′–3′ and 2′–3′ planes), were estimated using the scheme described in [33]. The resulting homogenized properties for the lamination sequence of shaft A and B are listed in Table 1.

### 3.2. Numerical Scheme

**Figure 4:**
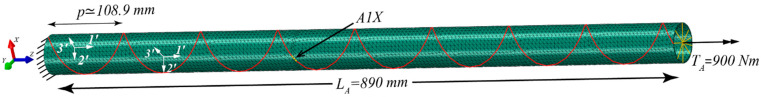
Finite element (FE) numerical model of shaft A with meshing pattern and applied boundary conditions.

Previous research studies have shown that when an optical fiber is embedded in CFRPs along the reinforcing fibers’ direction, the optical fiber is exposed to a quasi-homogeneous strain field [34], as demonstrated with finite element (FE) numerical modeling [35]. In this framework, a 3D representation of the tubular shafts A and B was created in Abaqus Standard v6.12 [36], implementing the homogenized material constants listed in Figure 1 in a linear elastic orthotropic material model and assuming also small displacements and linear global response. One edge of the shafts was fixed, while the maximum experimental torque per shaft was applied on the other edge via rigid body tie constraint and a control point, as illustrated in Figure 4. The material orientation was implemented in a discrete manner for every element so that the 1′–2′ properties refer always to the tangent to the circumference plane, and the 3′ direction to remain always normal to the shaft’s surface. 

Assuming that the Bragg reflection-peak shifts correspond to the normal axial strains applied on the optical fibers (birefringence and peak split are practically absent for the examined gratings), then the corresponding normal strains from the FE simulations need to be derived. Thus, a partition with the direction of the optical fibers with surface-mounted FBGs A1X and B1X (see Figure 2 for FBG nomenclature) was created on shafts’ geometry in order to facilitate the definition of the local coordinate system and consequently evaluate the local normal strains $\varepsilon_{z}$. The partition for optical fibers of FBG A1X was implemented using the equation of a helix (see also Figure 4) as follows: p=tanφ·D · π, where p is the pitch of the helix, φ is helix’s angle defined by the positive helical twist direction, i.e., $\varphi =90^{o}-\theta$ for θ the ply angle, and D is the external diameter. This helical partition allows only quadratic tetrahedral elements (Abaqus C3D10) to be employed. The global element’s edge length was 5 mm, while a secondary partition was created with elements of half size to average the strains along the corresponding gaging length (5 mm), using measurements of five nodes and two elements (the reader may also refer to Figure 10 in Section 4.2). The same meshing pattern was used on shaft B for uniformity, even-though the optical fiber was positioned in a circumferential manner. For shaft A, the model comprised ~30,300 elements and for shaft B ~41,300.

## 4. Results and Discussion

### 4.1. Optical Fiber Gratings Sensor Embedment and Residual Strains

**Figure 5:**
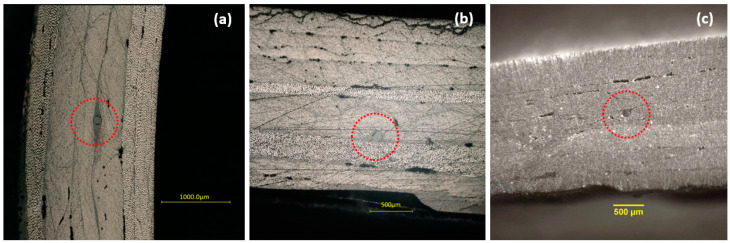
Microscope images of cross sections of the composite specimens at the location of the embedded fibers: (**a**) 0° FBG B2 (**b**) 55° FBG A1 (**c**) 90° FBG B1. The cross section of the optical fiber can be seen at the center of the red dotted circles. All sections were made at angles normal to the corresponding optical fiber direction.

After testing, cross sections of the shafts at the location of the embedded FBG were examined under a digital microscope (Keyence VHX5000) to verify the orientation of the optical fibers following the fabrication and curing process. Figure 5 depicts microscope images of the CRFP shafts’ cross section at the FBGs location for the three different embedded fiber orientations described in Figure 2. In each case, the composite specimen was cut and polished vertically to the optical fiber cross section. The microscopy reveals that the composite has low porosity and, moreover, that the optical fibers are well integrated within the material. Nevertheless, some realignment of the fibers is observed and attributed to the compaction during the curing process. Local thickness measurements on the sectioned samples gave values in the range of 1.9–2.2 mm, which verify the nominal cured ply thickness mentioned in Section 2. Related to the specific carbon fiber layer that each FBG fiber is embedded in and with regard to the layer sequence described in the Materials and Methods Section (i.e., ±55°, ±55°, +86°, ±55°, ±55°, +86°, ±55°, ±55°), the longitudinal FBG B2 (Figure 5a) lies within the fourth layer, the helical FBG A1 (Figure 5b) is at the outer edge of the fifth layer, while the circumferential FBG B1 (Figure 5c) is at the inner edge of the same layer.

**Figure 6:**
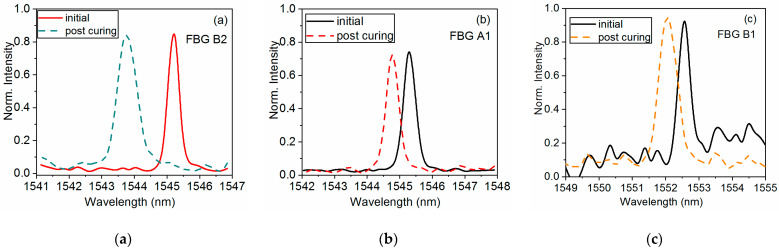
Reflected signal of embedded fiber Bragg gratings before and after resin curing for different fiber orientations with respect to the shaft axis (**a**) 0° FBG B2 (**b**) 55° FBG A1 (**c**) 90° FBG B1.

The spectral signal of the embedded FBGs was monitored after completion of the curing process and was compared to their initial spectral signature to examine the effect of embedment on the sensors. The results are appended in Figure 6, and all FBGs regardless of their orientation exhibit a blue-wavelength-shift after curing.

For the FBG placed parallel to the reinforcing fibers, the signal depicts a clear blue shift without shape deformations as shown in Figure 6b. For the other two orientations, the blue shift is accompanied by peak broadening due to non-uniform or non-axial residual stresses. Specifically, the bandwidth of FBG B2 is almost doubled (increased by 87%), while the broadening is smaller for FBG B1, with an increase of 46% observed in its full width at half maximum (FWHM).

**Figure 7:**
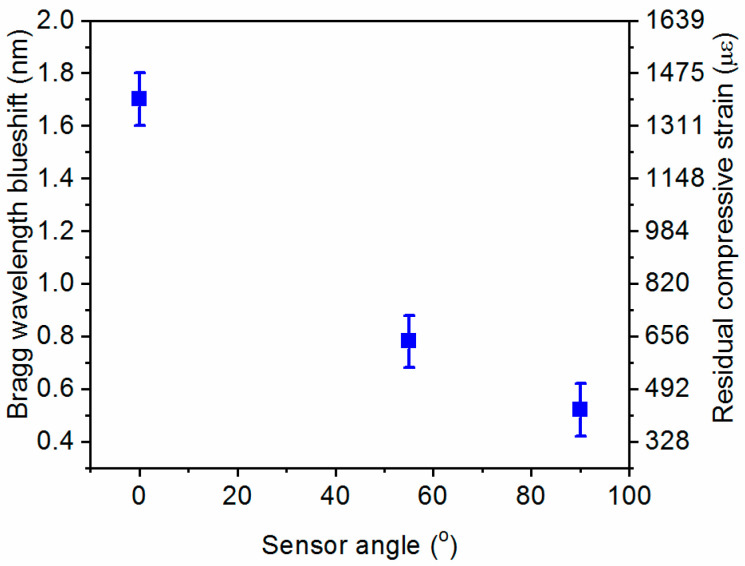
Bragg wavelength blue shift and corresponding residual compressive strain after thermal curing for different sensor orientations with respect to the shaft axis (0° indicates a sensor parallel to the shaft axis). The error bars represent the 0.2 nm hardware resolution of the spectrometer.

The recorded blue-wavelength-shift suggests compressive strain in the optical fibers due to the production process. This is in agreement with the verified size reduction of the shafts after curing and is mainly linked to the thermally induced shrinkage of the resin [37]. From our measurements, we can identify a trend between sensor orientation and recorded blue shift after curing as shown in Figure 7. The graph also depicts the corresponding residual normal compressive axial strain calculated from the measured Bragg wavelength shifts as described in Section 2. The sensor oriented along the axis of the shaft (0°) recorded the highest residual strain, while a sensor circled around the shaft (90°) experienced the smallest residual strain. This behavior is attributed to the effect of the reinforcing carbon fibers that have a very low thermal expansion coefficient and a significant orthotropic nature with a modulus in the longitudinal direction of at least one order of magnitude with respect to other directions. Further studies can utilize such data to predict the effect of the production process on the inevitable shrinkage of the composite structure after curing and its impact on structural durability. 

### 4.2. Torque Induced Strain Monitoring: Experimental and Numerical Results

**Figure 8:**
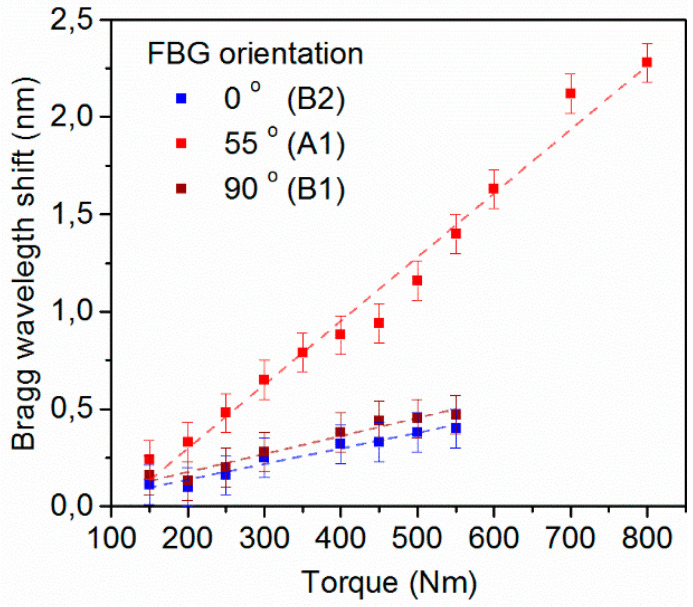
Bragg wavelength shift of embedded fiber Bragg gratings with different orientations for increasing applied torque (0° indicates sensors parallel to the shaft axis). The error bars represent the 0.2 nm hardware resolution of the spectrometer.

Shafts A and B were manually subjected to increasing levels of torque up to 800 Nm, as described in Section 2 (see also experimental set up in Figure 3). During the measurements, the metallic fittings of shaft B that allow attachment of the shaft with the torque wrench, detached at a torque level of 550 Nm, limiting the envelope of measurement for this shaft to a lower range. The recorded Bragg wavelength shifts for the three embedded FBGs are illustrated in Figure 8 and all gratings exhibit a positive linear wavelength shift. Based on the slope of the linear fit of Figure 8, the sensitivity for each sensor orientation is calculated. In particular, the grating embedded parallel to the carbon reinforcing fibers (55°) exhibits a sensitivity of 3.6 pm/Nm (with linear regression, R^2^: 0.977), while the sensitivities recorded for the other two orientations are indistinguishable from each other within the experimental error and of a lower value around 0.8–0.9 pm /Nm (R^2^: 0.948–0.956). As indicated from the results, the sensor oriented parallel to the carbon fibers wound at 55° exhibits the higher sensitivity in monitoring torque-induced strain. Specifically, the sensitivity recorded is more than 4× times higher than the other examined orientations. No significant variation in sensitivity was recoded between sensors placed longitudinally and circumferentially to the shaft. As analyzed later, this is mainly attributed to the fact that FBGs are calibrated to measure normal strains and not shear ones.

**Figure 9:**
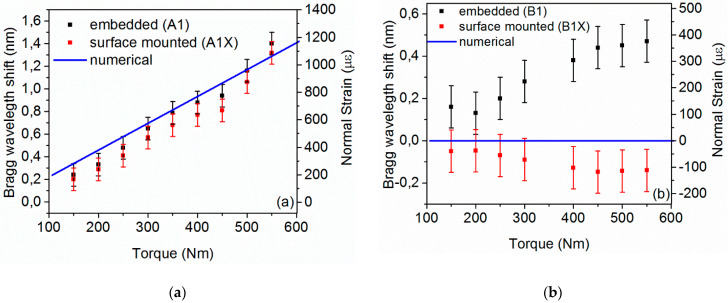
Bragg wavelength shift and corresponding normal strain for embedded (A1, B1) and surface-mounted (A1X, B1X) gratings under increasing torque; (**a**) FBGs at 55°, A1 and AX1 of shaft A and (**b**) FBG at 90°, B1 and B1X of shaft B. The error bars represent the 0.2 nm hardware resolution of the spectrometer.

As described in Section 2, gratings were surface mounted on the composite shafts, as depicted in Figure 2, to compare the response of embedded and surface sensors in strain monitoring due to torque. The recorded wavelength of the embedded (A1) and surface-mounted (A1X) FBGs with an orientation parallel to the carbon reinforcing fibers is illustrated in Figure 9a, while Figure 9b depicts the response of the circumferential sensors (B1 and B1X). The corresponding axial normal strain is also shown, calculated as described in Section 2. In each case, the experimental data of the surface-mounted sensors are compared with the numerical simulation based on the homogenized material model described in Section 3.1.

**Figure 10:**
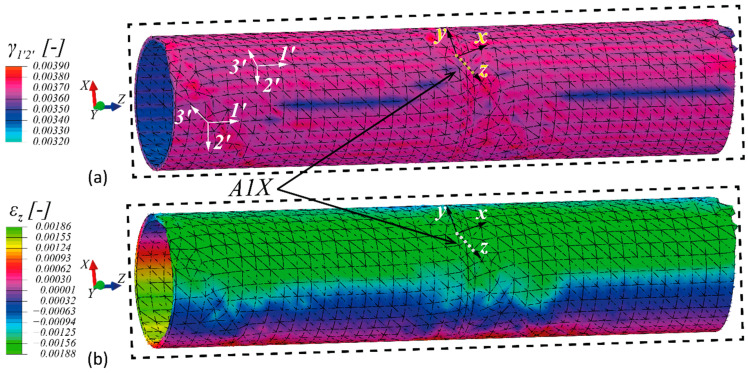
Strain profile resulted from the numerical FE simulations of shaft A for a torque of 900 Nm; (**a**) in-plane shear strains (at the discrete 1′–2′ planes tangent to the shaft), and (**b**) normal strains in the z-direction of the local coordinate system around the A1X FBG. The z-direction is tangent to the optical fiber at the A1X location.

Figure 10 shows the strain profiles obtained from the numerical simulation of shaft A. The calculated in-plane engineering shear strains’ profile, $\;\gamma_{1^{\prime }2^{\prime }}$ is shown in Figure 10a. Interestingly, the range of$\;\gamma_{1^{\prime }2^{\prime }}$ profile is very close to the minimum and maximum shear strain that can be predicted for an isotropic tube [38], of a shear modulus$,\;G=G_{1^{\prime }2^{\prime }}$ and a 900 Nm torque, i.e., ~3400 με, for the inner surface and 3700 με, for the outer one. It has to be noted that since the simulations are linear elastic, with linear geometry approximation the strains shall scale linearly with the increase of torque, as also seen in the experimental campaign (see Figure 8). The normal strain profile that corresponds to the local coordinate system of the surface-mounted A1X–FBG is shown in Figure 10b. Conventional strain transformation from the Cartesian 1′2′3′ coordinate system to the indicated local xyz that is tangent to the shaft at A1X–FBG position, illustrated in Figure 10b, was performed to extract the strain results that allow for direct comparison with the normal strains that were measured experimentally.

For an applied torque of 900 Nm and 550 Nm on shaft A and B, respectively, a rotation at the load application face of ~7.8° and 6.1°, respectively, is obtained from the numerical model. This leads to a rotational stiffness of ~115.4 Nm/degree, for shaft A and ~90.2 Nm/degree, for shaft B. Based on the numerical and experimental strains compared in Figure 9, the numerical scheme provided the same trend and range of strains for A1X. Regarding BX1, the model predicts very low strains. This is expected given that the optical fiber B1 is applied in a circumferential manner on the shafts; thus, it lies at the direction that pure shear stresses due to torque are expected, while the normal stresses should be essentially zero [38].

FBGs in general, respond to axial strains with simple peak shifts, while complex states are depicted by peak splits and birefringence [34]. When an isotropic body is exposed to pure in-plane shear, the principal normal stresses occur at a 45° inclined plane. This explains why the measurements at A1X are significantly highear (55° inclined plane), compared to B1X (90° inclined plane, maximum shear stress plane). Nevertheless, the layered structure of the shafts reveals that the internal normal stresses, even at the maximum shear plane, may be different from zero (see B1 in Figure 9b). This may be attributed to the different directions of the Poisson’s effect per ply that creates interlaminar shear stresses [29].

Useful measurements can thus be obtained when the optical fibers in the embedding scheme are oriented parallel to the reinforcing fibers to avoid major disturbance of their directionality and provide optimal strain transfer from the composite material to the optical fiber. Preferably, this orientation would also lie as close as possible to the principal stress plane. The exact stress state in a given ply may be defined by an inverse scheme in which normal strains from FBG measurements can be utilized to evaluate the stresses that can cause potential failure. This can be the content of future studies.

As indicated from the results presented in the preceding sections, surface-mounted FBGs along the reinforcing fiber direction can constitute a viable option for the measurement of torque-induced strain, albeit requiring a manual, post-winding installation process. While embedded FBGs can capture residual strains from the different stages of the manufacturing process and leave the outer surface of the shaft intact, they require laborious installation and precise alignment with respect to the composite structure. Therefore, the decision on the use of embedded or surface-mounted sensors should be based on factors such as ease of installation, protection, and long-term stability of the sensor, in addition to any particular application requirements (e.g., delamination monitoring), favoring correspondingly one of the two approaches. 

## 5. Conclusions

We report on the use of optical fiber Bragg gratings sensors for torque-induced strain monitoring in filament-wound composite cylindrical structures that find application as torque transmission shafts and other types of axle structures. Our study involved embedded and surface-mounted optical fiber gratings and examined the response of gratings, with different orientations with respect to the shaft’s axis, under static torsion. Experimental results, supported by numerical simulations, showed that maximum sensitivity, equal to 3.6 pm/Nm, is recorded for sensors aligned with the reinforcing carbon fibers. Additionally, surface-mounted gratings of the same orientation constitute a viable option for the measurement of torque-induced strain. Moreover, experimental results and numerical analysis demonstrated that FBG sensors can contribute to SHM of shear-dominated load cases (e.g., torque load) as long as they are employed in appropriate directions. The aforementioned findings contribute to the implementation of SHM of CFRP parts, subjected to torque loads that can be applied during periodic inspections, under static conditions, or even for real-time measurements using commercially available fiber optic rotary joints. Further studies are required for the direct implementation of SHM.

## Figures and Tables

**Table 1:** Engineering constants of (i) homogenized unidirectional (UD) lamina (material principal directions: 1–2–3), derived by analytical micromechanical formulation, and (ii) homogenized laminate (flat laminate directions: 1′–2′–3′) shafts A and B using classical laminate theory (CLT) for the in-plane properties (1′–2′) and Kress’s model [33] for the through-thickness ones (1′–3′ and 2′–3′), shown in parenthesis.

	$E_{11}\;[\mathrm{GPa}]$	$E_{22}\;[\mathrm{GPa}]$	$E_{33}\;[\mathrm{GPa}]$	$\nu_{12}\;[-]$	$\nu_{13}\;[-]$	$\nu_{23}\;[-]$	$G_{12}\;[\mathrm{GPa}]$	$G_{13}\;[\mathrm{GPa}]$	$G_{23}\;[\mathrm{GPa}]$
UD-lamina	133.5	7.4	7.4	0.31	0.31	0.43	3.6	3.6	2.4
	$E_{1^{\prime }}$ [GPa]	$E_{2^{\prime }}$ [GPa]	$E_{3^{\prime }}$ [GPa]	$\nu_{1^{\prime }2^{\prime }}$ [-]	$\nu_{1^{\prime }3^{\prime }}$ [-]	$\nu_{2^{\prime }3^{\prime }}$ [-]	$G_{1^{\prime }2^{\prime }}$ [GPa]	$G_{1^{\prime }3^{\prime }}$ [GPa]	$G_{2^{\prime }3^{\prime }}$ [GPa]
[±55, ±55, +86, ±55]_s_	16.1	55.5	(8.4)	0.49	(0.29)	(−0.12)	36.3	(2.6)	3.1

## Data Availability

The data presented in this study are available on request from the corresponding author.

## References

[1] Chung D.D.L. (2010). Composite Materials: Science and Applications.

[2] Shen F.C. (1995). A filament-wound structure technology overview. Mater. Chem. Phys..

[3] Ferri Aliabadi M.H., Sharif Khodaei Z. (2017). Structural Health Monitoring for Advanced Composite Structures.

[4] Measures R.M. (2001). Structural Monitoring with Fiber Optic Technology.

[5] Kang H.-K., Kang D.-H., Bang H.-J., Hong C.-S., Kim C.-G. (2002). Cure monitoring of composite laminates using fiber optic sensors. Smart Mater. Struct..

[6] Geernaert T., Luyckx G., Voet E., Nasilowski T., Chah K., Becker M., Bartelt H., Urbanczyk W., Wojcik J., Waele W.D. (2009). Transversal Load Sensing with Fiber Bragg Gratings in Microstructured Optical Fibers. IEEE Photonics Technol. Lett..

[7] Sulejmani S., Sonnenfeld C., Geernaert T., Luyckx G., Van Hemelrijck D., Mergo P., Urbanczyk W., Chah K., Caucheteur C., Mégret P. (2013). Shear stress sensing with Bragg grating-based sensors in microstructured optical fibers. Opt. Express.

[8] Sonnenfeld C., Luyckx G., Sulejmani S., Geernaert T., Eve S., Gomina M., Chah K., Mergo P., Urbanczyk W., Thienpont H. (2015). Microstructured optical fiber Bragg grating as an internal three-dimensional strain sensor for composite laminates. Smart Mater. Struct..

[9] De Pauw B., Goossens S., Geernaert T., Habas D., Thienpont H., Berghmans F. (2017). Fibre Bragg Gratings in Embedded Microstructured Optical Fibres Allow Distinguishing between Symmetric and Anti-Symmetric Lamb Waves in Carbon Fibre Reinforced Composites. Sensors.

[10] Canal L.P., Sarfaraz R., Violakis G., Botsis J., Michaud V., Limberger H.G. (2014). Monitoring strain gradients in adhesive composite joints by embedded fiber Bragg grating sensors. Compos. Struct..

[11] Goossens S., De Pauw B., Geernaert T., Salmanpour M.S., Sharif Khodaei Z., Karachalios E., Saenz-Castillo D., Thienpont H., Berghmans F. (2019). Aerospace-grade surface mounted optical fibre strain sensor for structural health monitoring on composite structures evaluated against in-flight conditions. Smart Mater. Struct..

[12] Nascimento M., Inácio P., Paixão T., Camacho E., Novais S., Santos T.G., Fernandes F.M.B., Pinto J.L. (2020). Embedded Fiber Sensors to Monitor Temperature and Strain of Polymeric Parts Fabricated by Additive Manufacturing and Reinforced with NiTi Wires. Sensors.

[13] Hernández-Moreno H., Collombet F., Douchin B., Choqueuse D., Davies P., González Velázquez J.L. (2009). Entire Life Time Monitoring of Filament Wound Composite Cylinders Using Bragg Grating Sensors: II. Process Monitoring. Appl. Compos. Mater..

[14] Hernández-Moreno H., Collombet F., Douchin B., Choqueuse D., Davies P. (2009). Entire Life Time Monitoring of Filament Wound Composite Cylinders Using Bragg Grating Sensors: III. In-Service External Pressure Loading. Appl. Compos. Mater..

[15] Kang H.-K., Park J.-S., Kang D.-H., Kim C.-U., Hong C.-S., Kim C.-G. (2002). Strain monitoring of a filament wound composite tank using fiber Bragg grating sensors. Smart Mater. Struct..

[16] Park S.W., Kang D.H., Bang H.J., Park S.O., Kim C.G. (2006). Strain Monitoring and Damage Detection of a Filament Wound Composite Pressure Tank Using Embedded Fiber Bragg Grating Sensors. Key Eng. Mater..

[17] Ravet F., Zou L., Bao X., Chen L., Huang R.F., Khoo H.A. (2006). Detection of buckling in steel pipeline and column by the distributed Brillouin sensor. Opt. Fiber Technol..

[18] Yang W., Tavner P.J., Crabtree C.J., Feng Y., Qiu Y. (2014). Wind turbine condition monitoring: Technical and commercial challenges. Wind Energy.

[19] Hashimoto M., Kiyosawa Y., Paul R.P. (1993). A torque sensing technique for robots with harmonic drives. IEEE Trans. Robot. Autom..

[20] Gao C., Wang D., Yu Y., Zheng Y., Jiang S., Deng Z. (2015). Measurement technique of mechanical parameters for tubular workpiece based on Fiber Bragg Grating. Chin. J. Appl. Mech..

[21] Leal-Junior A.G., Frizera A., Marques C., Sánchez M.R.A., Botelho T.R., Segatto M.V., Pontes M.J. (2018). Polymer optical fiber strain gauge for human-robot interaction forces assessment on an active knee orthosis. Opt. Fiber Technol..

[22] Leal-Junior A.G., Theodosiou A., Min R., Casas J., Díaz C.R., Santos W.M.D., Pontes M.J., Siqueira A.A.G., Marques C., Kalli K. (2019). Quasi-Distributed Torque and Displacement Sensing on a Series Elastic Actuator’s Spring Using FBG Arrays Inscribed in CYTOP Fibers. IEEE Sens. J..

[23] Leal-Junior A.G., Frizera A., Marques C., Sánchez M.R.A., Santos W.M.d., Siqueira A.A.G., Segatto M.V., Pontes M.J. (2018). Polymer Optical Fiber for Angle and Torque Measurements of a Series Elastic Actuator’s Spring. J. Lightwave Technol..

[24] Sanchez M.R.A., Leal-Junior A.G., Segatto M.V., Marques C., dos Santos W.M., Siqueira A.A.G., Frizera A. (2018). Fiber Bragg grating-based sensor for torque and angle measurement in a series elastic actuator’s spring. Appl. Opt..

[25] Konstantaki M., Childs P., Sozzi M., Pissadakis S. (2013). Relief Bragg reflectors inscribed on the capillary walls of solid-core photonic crystal fibers. Laser Photonics Rev..

[26] Jülich F., Aulbach L., Wilfert A., Kratzer P., Kuttler R., Roths J. (2013). Gauge factors of fibre Bragg grating strain sensors in different types of optical fibres. Meas. Sci. Technol..

[27] Leal-Junior A., Frizera A., Marques C. (2020). Development and Characterization of UV-Resin Coated Fiber Bragg Gratings. Sensors.

[28] Sarkar S., Tarhani M., Eghbal M.K., Shadaram M. (2020). Discrimination between strain and temperature effects of a single fiber Bragg grating sensor using sidelobe power. J. Appl. Phys..

[29] Daniel I., Ishai O. (1994). Engineering Mechanics of Composite Materials.

[30] Dow Chemical Company VORAFORCE™ Filament Winding, Epoxy Resin System-VORAFORCE TW 100 Series. https://www.dow.com.

[31] TORAYCA^®^. T700S Standard Modulus Carbon Fiber. https://www.toraycma.com.

[32] Kriz R.D., Stinchcomb W.W. (1979). Elastic moduli of transversely isotropic graphite fibers and their composites. Exp. Mech..

[33] Kress G. Three-Dimensional Properties of a Generally Orthotropic Symmetric Laminate. Proceedings of the European Mechanics Colloqium 214.

[34] Botsis J., Nicolais L. (2012). Fiber Bragg Grating Applied to In Situ Characterization of Composites. Wiley Encyclopedia of Composites.

[35] Stutz S., Cugnoni J., Botsis J. (2011). Crack—Fiber sensor interaction and characterization of the bridging tractions in mode I delamination. Eng. Fract. Mech..

[36] (2012). Abaqus Analysis User’s Manual (v6.12).

[37] Kravchenko O.G., Kravchenko S.G., Pipes R.B. (2016). Chemical and thermal shrinkage in thermosetting prepreg. Compos. Part A Appl. Sci. Manuf..

[38] Timoshenko S., Goodier J.N. (1951). Theory of Elasticity.

